# Differences in Telomere Length between Adolescent Females with Anorexia Nervosa Restricting Type and Anorexia Nervosa Binge-Purge Type

**DOI:** 10.3390/nu15112596

**Published:** 2023-06-01

**Authors:** Orit Uziel, Hadar Dickstein, Einat Beery, Yael Lewis, Ron Loewenthal, Eran Uziel, Zipi Shochat, Abraham Weizman, Daniel Stein

**Affiliations:** 1 Felsenstein Medical Research Center, Sackler School of Medicine, Tel Aviv University, Petah Tikva 69978, Israel; oritu@clalit.org.il (O.U.);; 2 Sackler School of Medicine, Tel Aviv University, Tel Aviv 69978, Israel; 3 Safra Children’s Hospital, Sheba Medical Center, Ramat Gan 52621, Israel; 4 Shalvatah Mental Health Center, Hod Hasahron 45100, Israel; 5 Research Unit, Geha Mental Health Center, Petah Tikva 49100, Israel; 6 Statistical Service, Rabin Medical Center, Petah Tikva 49100, Israel

**Keywords:** adolescence, anorexia nervosa, eating disorders, telomere length

## Abstract

Physiological and psychological distress may accelerate cellular aging, manifested by shortening of telomere length (TL). The present study focused on TL shortening in anorexia nervosa (AN), an illness combining physiological and psychological distress. For that purpose, we measured TL in 44 female adolescents with AN at admission to inpatient treatment, in a subset of 18 patients also at discharge, and in 22 controls. No differences in TL were found between patients with AN and controls. At admission, patients with AN-binge/purge type (AN-B/P; *n* = 18) showed shorter TL compared with patients with AN-restricting type (AN-R; *n* = 26). No change in TL was found from admission to discharge, despite an improvement in body mass index standard deviation score (BMI-SDS) following inpatient treatment. Older age was the only parameter assessed to be correlated with greater TL shortening. Several methodological changes have to be undertaken to better understand the putative association of shorter TL with B/P behaviors, including increasing the sample size and the assessment of the relevant pathological eating disorder (ED) and non-ED psychological correlates in the two AN subtypes.

## 1. Introduction

Anorexia nervosa (AN) is a severe, potentially life-threatening psychiatric disorder with a lifetime prevalence of 0.80% to 3.60% [1,2]. AN is associated with a restriction of food intake due to an irrational fear of being overweight and a relentless desire for thinness, leading to intentional dieting behaviors and a significantly low body weight alongside considerable body image disturbances [3].

AN mainly affects female adolescents and young adults [3]. Although 70–80% may remit over time [2,3], around 20% of patients may show a protracted severe course of illness [4,5]. AN is associated with numerous medical complications that are mostly directly attributable to malnutrition and weight loss [6,7]. It presents with the highest mortality rate of all psychiatric disturbances [8]. Almost every organ system can be affected during the course of the illness [6,7]. The most prominent risks of AN-associated malnutrition relate to alterations in cardiac functioning—specifically, prolongation of the corrected QT (QTc) interval, various blood dyscrasias, osteoporosis, and neurocognitive dysfunction, likely associated with gray and white matter changes [6,7,9]. Many hormonal changes may occur in AN, including hypothyroxinemia, hypercortisolemia, changes in cholesterol metabolism, hypogonadism, increased insulin resistance, and diminished growth hormone activity, resulting in decreased secretion of insulin-like growth factor I and growth hormone resistance [6,10,11]. Whereas many medical disturbances may resolve with weight restoration, some may persist in remitted patients, including neurocognitive disturbances, osteoporosis, and problems with fertility [6,7].

### 1.1. Telomere Dynamics in AN

The medical disturbances involved in AN and the chronic course of the illness within a significant minority of the patients raise the issue of the potential impact of protracted malnutrition and other illness-related disturbances—e.g., purging behaviors or hormonal changes—on the dynamics of telomeres. Cellular telomeres are repetitive sequences of (TTAGGG) 1000-2000, located at the end of each human chromosome. The length of the telomeres (TL) gradually shortens with each DNA replication accompanying cell division [12]. This gradual shortening signals the cells to enter an irreversible state called replicative senescence, expressed in humans as ageing [12,13,14,15].

There is a link between shorter TLs and age-related physiological disturbances, including arthritis, hypertension, diabetes mellitus, and cardiovascular illness, as well as between shorter TLs and early mortality [16,17,18]. In addition to physical stress, mental stress including that found in post-traumatic stress disorder (PTSD), anxiety disorders, depression, and schizophrenia [19,20,21,22,23,24,25] may also lead to TL shortening. This is because TL shortening has been suggested to serve as a marker of cumulative exposure to stress, which may be considered a risk factor of depressive and anxiety disorders [19]. Indeed, one study [19] found that among women, those with generalized anxiety disorder and panic disorder have shorter TLs than those with no anxious affect. Another study has shown that individuals with a history of major depression have shorter TLs relative to controls and that the severity and duration of depression are inversely correlated with TL [21]. The potential molecular mechanisms associated with TL shortening in various psychiatric illnesses include oxidative stress, inflammatory processes, and mitochondrial dysfunction linking [25]. Yet, it is unclear whether psychological distress is a cause and/or a consequence of TL shortening [25].

Several studies have found that overweight, obesity, and metabolic dysregulation may increase the risk for TL shortening [26,27]. Therefore, it is plausible that undernutrition, such as that found in AN, can also be associated with TL shortening [28,29,30]. Moreover, some studies [31,32,33], although not all [34], have found an association between elevated cholesterol levels [31] and thyroid hormones abnormalities [32,33] with TL shortening. This is of note in AN since malnutrition is associated with a reduction in triiodothyronine [35,36], which, in turn, might be associated with low catabolism [36] and, hence, elevated levels of cholesterol [35,36,37,38]. Second, the hypercholesterolemia in patients with AN may be associated also with a secondary synthesis [39] linked to hyperglycemia, consequent of increased cortisol levels associated with weight reduction [6,7].

In addition, patients with AN may experience considerable mental stress and elevated rates of comorbid psychiatric disorders such as depressive disorders, anxiety disorders, and PTSD [40,41,42,43,44,45]; this can add to the increased risk of TL shortening in these patients [19,20,21,22,25].

To the best of our knowledge, TL shortening was not studied in AN. In the current study, we sought to assess the putative association of malnutrition and pathological eating behaviors in AN with TL shortening, and to clarify whether TL shortening would be attenuated by post-treatment weight restoration and an improvement in disordered eating.

Second, there are two subtypes of AN. Restrictive-type AN (AN-R) is characterized by engaging in intentional restriction of food and increased physical activity. The binge–purge subtype (AN-B/P) is characterized by additional engaging in purging behaviors—i.e., self-induced vomiting and misuse of laxatives, diuretics, or enemas—and/or in bingeing, not seldom resulting from the patients’ restrictive and/or purging behaviors [46,47,48]. The AN-B/P type is considered the more severe variant, often involving more severe ED pathology, greater rates of comorbid depression, anxiety, obsessive compulsive disorder, PTSD, substance use disorders, and an overall worse prognosis [40,41,42,43,44,45]. Thus, it is interesting to assess whether two types of AN would differ in the shortening of TL.

### 1.2. Current Study Hypotheses

Based on the above findings, our hypotheses are as follows:

1. Hospitalized adolescent girls with AN would show shorter TLs in comparison to control participants.
2. TL shortening would be greater in patients with AN-B/P vs. patients with AN-R.
3. At discharge from inpatient treatment, TL would be greater than at admission in both subtypes of AN.
4. Baseline TL would be associated with demographic (age, age at illness onset, duration of illness) and baseline medical parameters body mass index standard deviation score—BMI-SDS, and cholesterol and thyroid hormone levels, see [31,32,33].
5. The change in TL in patients with AN from admission to discharge would be associated with the baseline parameters noted in Hypothesis 4, as well as with the duration of inpatient treatment and the change in the medical parameters from admission to discharge.

## 2. Materials and Methods

### 2.1. Participants

The study included 44 inpatients diagnosed with AN and 22 control participants. Inclusion criteria comprised female sex, age of 13–19 years, and good knowledge of the Hebrew language. Exclusion criteria were lifetime or current diagnosis of organic brain disorder, bipolar disorder, schizophrenia spectrum disorders, substance use disorders, intellectual disability, physical disorders that can affect eating or weight (e.g., diabetes mellitus or thyroid disorders), and use of medications for a period longer than four consecutive weeks.

The 44 inpatients with AN were recruited between 1 January 2015 and 31 December 2018 from the adolescent inpatient eating disorders (ED) department at Safra Children’s Hospital in Sheba Medical Center, Tel Hashomer, Israel. The AN diagnosis and subtype were established using a semi-structured interview based on the DSM 5 [48] criteria, yielding 26 patients with AN-R and 18 with AN-B/P.

Control participants were a convenience sample of female high-school students matched by residential area to the inpatient group with AN. The controls were recruited by the research team. To determine their health status, controls completed open-ended questions about history of significant physiological disorders, emotional disorders, and chronic medication use. Further, an experienced psychiatrist administered the SCOFF questionnaire [49] to the control participants (the acronym SCOFF was created from the first letters of its questions [49]). The SCOFF questionnaire includes five yes/no questions tapping different aspects of Eds, namely, losing weight, feeling fat despite others’ claiming otherwise, feeling lack of control over eating, feeling that food controls one’s life, and self-induced vomiting when feeling full. The Hebrew translation of the SCOFF was validated in Israeli community participants [50]. A total SCOFF score of ≥2 points was found to be 100% sensitive and 87.5% specific for the detection of EDs in the community [49]. The present study included only controls answering negatively to the open-ended history items and to all five SCOFF items. Fifteen controls were excluded for not fulfilling these criteria.

### 2.2. Procedure

All participants, and parents or legal guardians of minors under age 18, provided written informed consent after receiving an explanation of the study’s goals and methodology. The study was approved by the Helsinki committee of Sheba Medical Center (Number 2504-15-SMC).

Using semi-structured interviews, experienced child and adolescent psychiatrists established the inpatients’ AN diagnosis and subtype based on the DSM 5 [48] criteria. Patients were excluded from study participation if (a) their AN diagnosis and subtype were not confirmed unanimously in clinical team meetings of the inpatient department or (b) they were diagnosed with atypical AN according to the DSM 5 criteria [48].

Relevant demographic and clinical data were collected from the patients’ electronic medical records. A subset of 18 inpatients with AN (41% of the original inpatient sample) completed the study and additionally provided post-treatment data at the time of discharge from inpatient treatment. The 26 patients not participating at the post-treatment interval either refused to continue with the study (*n* = 19) or were discharged from the department before completing their treatment (*n* = 7). Mean duration of hospitalization for these 18 patients was 4.24 months (SD = 2.3). In this department, patients with AN-R are discharged after reaching their required target weight and maintaining it for at least two consecutive weeks. The decision about the target weight in this department is based on the patients’ premorbid weight and height percentiles. Patients with AN-B/P are additionally required to refrain from B/P behaviors for at least two consecutive weeks before discharge.

### 2.3. Data Collection

#### 2.3.1. Clinical Data

Data about age, weight, and height were collected from the inpatients’ electronic medical records at admission (*n* = 44) and at discharge (*n* = 18). Patients in this department are weighed once weekly, in the morning hours before their first meal, according to a standardized procedure [51]. Height is measured once monthly. BMI is calculated as weight in kilograms divided by height in square meters [52]. Further, we calculated the patients’ BMI standard deviations score (BMI-SDS) because of their young age. In this study, we related the weight and height taken at admission to and discharge from inpatient treatment.

#### 2.3.2. Cholesterol and Thyroid Hormones Testing

Blood samples for cholesterol levels and thyroid function tests are collected routinely in this inpatient department once monthly between 07:00 a.m. and 09:00 a.m., following an overnight fast. Thyroid function tests include the following: thyroid stimulating hormone (TSH), thyroxine (T4), and triiodothyronine (T3). In this study, we related the cholesterol and thyroid hormone measurements taken at admission to and discharge from inpatient treatment.

#### 2.3.3. Blood Sampling for TL Measurement

Inpatients who agreed to participate in the study provided an additional 10 mL blood sample for TL assessment at the time of admission (*n* = 44) and at the time of discharge from inpatient treatment (*n* = 18). Blood samples were stored in the HLA-typing laboratory at the Sheba Medical Center.

DNA isolation was performed at Felsenstein Medical Research Center, Rabin Medical Center, and Sackler Faculty of Medicine, Tel Aviv University. Extraction of DNA from each 10 mL blood sample was conducted using the Gentra PureGene DNA Isolation kit (Qiagen, Hilden, Germany) based on the salting out procedure and following the manufacturer’s instructions.

#### 2.3.4. TL Measurement

TL was measured using a polymerase chain reaction (PCR) based method. Ten mL of peripheral blood was obtained from each participant. Red blood cells were lysed by using red blood cells lysis reagent (Biological industries, Beit Haemek, Israel) according to the provided manual. Basically, we incubated the blood samples with two volumes of red blood cells lysis solution for 10 min at room temperature to allow their lysis and subsequently centrifuged the samples for 3 min at 2500 rounds per minute (RPM) to precipitate the collected leukocytes. This step was repeated following resuspension of the leukocytes pellet with 1 ml PBS buffer. To isolate the DNA, we used the DNA isolation kit for mammalian blood (Roche, Basel, Switzerland) according to the provided protocol. Based on the salting out principle, this method enables long-term storage (up to two years) of the obtained leukocyte for later DNA isolation. For this purpose, the sample leukocytes were mixed with the white blood cell lysis buffer provided by the kit and stored at 4 °C until the DNA isolation procedure took place upon completion of the participants’ recruitment. To isolate the DNA from the lysed leukocytes, we thoroughly mixed them with the Protein Precipitation Solution provided by the kit. After precipitating the proteins and other debris by centrifugation, the DNA was further precipitated by ethanol precipitation and its concentration was measured using a nanodrop device (ThermoFisher Scientific, Waltham, MA, USA).

TL lengths were assessed by carrying out a modified technique based on Cawthon’s Quantitative PCR method [53] using the Absolute Human Telomere Length Quantification qPCR Assay Kit (ScienceCell, Carlsbad, CA, USA), which enabled the measurement of TLs in absolute base pair units. Each DNA sample was analyzed by two sets of primers provided by the kit: two primers for telomere sequences and two primers for a single copy reference gene. A reference value was used to calculate absolute TLs in base pair units. All reactions were carried out using the ABI Step One device (Life Technologies Corporation, Carlsbad, CA, USA).

Controls were not assessed for weight, height, cholesterol levels, and thyroid function tests. A blood sample to determine TL (10 mL) was collected only once from the control participants in the human leukocyte antigen (HLA) typing laboratory of Sheba Medical Center, Tel Hashomer, Israel.

### 2.4. Statistical Analysis

Independent *t* tests for between group differences were used in the comparisons of patients with AN and controls, and patients with AN-R vs. AN-B/P. Dependent *t* tests for within-group differences were used in the comparisons of admission vs. discharge data of the entire AN sample. We used the Wilcoxon signed-rank test in the comparison of the admission and discharge data of each AN subtype separately, because of deviation from normality. Analysis of covariance was used to control for a possible influence of age on TL shortening, as greater TL shortening is known to be associated with older age [18]. Pearson’s correlation coefficients were used to assess the correlations of TL with age and age at onset of AN; duration of illness at admission; and baseline levels of weight, height, BMI, BMI-SDS, cholesterol levels, and thyroid function tests. In addition, we used Pearson’s correlation coefficients to assess the correlations of the change in TL from admission to discharge with the aforementioned baseline parameters, duration of inpatient treatment, and the change from admission to discharge in the clinical and laboratory parameters. Statistical analyses were performed using IBM SPSS v.24 (Armonk, NY, USA) with significance set at *p* ≤ 0.05.

## 3. Results

Table 1 summarizes the differences between patients with AN and controls. Patients with AN were significantly younger than control participants. TL analysis showed no significant differences between patients with AN and controls.

In the AN group, the mean age of onset of AN was 13.16 years (SD = 1.30) and the mean duration of illness before admission was 2.24 years (SD = 1.54). At the time of admission, the mean weight of the patients with AN was 44.56 kgs (SD = 5.91), mean height was 159.76 m (SD = 6.35), mean BMI was 17.54 (2.15) kg/m^2^, and mean BMI-SDS was −1.14 (SD = 1.10). Regarding the admission laboratory tests, the mean cholesterol level was 165.5 mg/dL (SD = 23.45), mean TSH level was 2.45 mμ/L (SD = 1.29), mean T4 level was 9.77 pmol/L (SD = 1.54), and mean T3 level was 5.03 pmol/L (SD = 0.85). All laboratory tests were within normal ranges.

Table 2 summarizes the differences between patients with AN-R vs. AN-B/P. No significant differences in age, age of onset of AN, or BMI-SDS were found between patients in the two AN subtypes. Although significant between-group differences were found for BMI, we decided to relate to BMI-SDS and not to BMI in our analyses, as BMI-SDS is considered to be more pertinent for adolescents, taking into consideration the influence of age on BMI. Last, patients with AN-B/P had significantly shorter TLs than patients with AN-R, also after correction by age.

Laboratory tests at admission showed no significant differences between the two AN subtypes on cholesterol or thyroid levels, which were all within normal ranges.

Table 3 summarizes the differences between admission and discharge data for the subset of 18 patients with AN completing the study. No differences were found for TL, despite the patients’ significant improvement in BMI and BMI-SDS from admission to discharge. No significant change was found in cholesterol and thyroid hormones levels, which were within normal ranges at both intervals.

Table 4 describes the differences in TL, BMI, BMI-SDS, and laboratory data in the subset of two AN subtypes assessed at both admission and discharge. No between-group differences in TL were found in this subset at admission and at discharge, and no change was found in TL in both the AN-R and AN-B/P groups from admission to discharge (*p* = 0.42 and *p* = 0.22, respectively). Regarding the patients’ BMI and BMI-SDS, we found a significant improvement from admission to discharge in the AN-R group (*p* = 0.002 for both measures) but not the AN-B/P group (*p* = 0.16 and *p* = 0.13, respectively). The BMI and BMI-SDS of the AN-B/P group were greater than those of the AN-B/P group at admission (*p* = 0.01 and *p* = 0.04, respectively) but not at discharge (*p* = 0.99 and *p* = 0.48, respectively).

Table 5 presents the correlations of baseline TL with the different demographic and baseline clinical and laboratory variables included in the study. Patients’ age was the only variable significantly correlated with baseline TL (r = −0.308, *p* < 0.027), where older age was associated with shorter TL. Second, the change in TL from admission to discharge in the 18 patients assessed at both time points was not correlated with the demographic and baseline clinical and laboratory variables introduced, including age, and with the change in the clinical and laboratory parameters during inpatient treatment.

## 4. Discussion

The aim of this research was to assess TL in acutely-ill adolescent females with AN at admission to and at discharge from inpatient treatment, following weight restoration and improvement in maladaptive ED behaviors. Our findings show that Hypothesis 1 was not confirmed, in that no differences in TL shortening were found between patients with AN and control participants. This non-significant outcome might result from the relatively small number of participants included in the study. Second, the relatively short duration of illness in our adolescent patients with AN (M = 2.24 years, SD = 1.54) was insufficient to lead to TL shortening. Third, the AN group’s mean BMI (17.54 (2.15) kg/m^2^) and mean BMI-SDS (−1.14 (SD = 1.10)) at admission were likely not considered low enough to induce TL shortening. It is of note that in this adolescent inpatient department, patients with AN were hospitalized not only because of low weight accompanied by severe medial disturbances. Other indications for hospitalization included acute suicidal risk, inability of the family to cooperate with outpatient treatment, and failure of previous ambulatory and day-center interventions. 

An important finding of this study relates to the significantly greater TL shortening in patients with AN-B/P vs. AN-R, thus confirming Hypothesis 2. Prior research suggests that the mental stress characterizing PTSD, anxiety disorders, and depression—all appearing more frequently in the AN-B/P type [40,41,42,43,44,45]—can potentially lead to TL shortening [19,20,21,22,25]. Nonetheless, our study cannot confirm these suggestions as we have not compared the two AN subtypes on any psychometric scale and have not assessed psychiatric comorbidity. Future studies evaluating TL in the different subtypes of AN should assess psychiatric comorbidities and include relevant psychometric scales previously used for individuals with AN, e.g., the Eating Disorder Inventory-3 [54] or the Symptom Checklist-90-Revised test [55,56].

From a physiological perspective, patients with AN-R may manifest a higher frequency of underweight-mediated medical complications, including greater bone marrow dysfunction, hepatic dysfunction, and hypoglycemia [57]. Indeed, starvation has been repeatedly linked to TL shortening [58], and previous studies have shown lower BMI in patients with AN-R vs. AN-B/P [59,60]. However, in the present study, there were no differences in BMI-SDS between patients with AN-R and AN-B/P, likely because of the relatively short illness duration and the relatively less compromised BMI-SDS in our adolescent sample.

The finding of shorter TL in patients with AN-B/P vs. AN-R in the present study is, thus, not related to differences in age or BMI-SDS. One putative explanation for the difference between the two subtypes is that electrolyte (natrium/potassium) and acid/base imbalance are more severe in patients with AN-B/P type because of their purging behaviors [6]. The association of this imbalance with TL shortening in AN-B/P type has not been studied. However, extrapolating from studies in other populations shows that the metabolic acidosis seen in people with age-related renal dysfunction may lead to greater shortening of TL [61,62]. One should bear in mind, in this respect, that laxative abuse in patients with AN-B/P might lead to metabolic acidosis [6].

Alternatively, a parallel hypothesis suggests that caloric restriction—reduction of food intake while avoiding malnutrition—may be an important intervention to attenuate telomere erosion associated with aging [63]. In fact, caloric restriction has been found to synergize with telomerase reverse transcriptase over-expression in increasing “health span” and extending longevity in old mice but not in young ones [64]. However, the findings of this study do not support this hypothesis, as the higher BMI in patients with AN-B/P [59,60] who show shorter TL suggests their condition to be closer to restriction (vs. malnutrition) than that of patients with AN-R.

The findings for Hypothesis 3, i.e., putative improvement in TL shortening occurring during inpatient treatment, and putative between-group difference in this improvement, were negative. This is likely associated with the high attrition percentage (59%) in our study, leading to a very small number of patients assessed at both time points. In addition, the lack of change in TL between admission and discharge, despite an improvement in BMI and BMI-SDS, can be related to the time of treatment being probably too short to induce changes in TL (mean duration of hospitalization is 4.24 months).

Hypotheses 4 and 5 have not been confirmed, as seen in the non-significant associations found between baseline TL, as well as between the change in TL from admission to discharge, and the other investigated parameters. One exception is the significant association found between shorter baseline TL and older age. Although TL’s link with age is plausible for individuals at older ages [18], it is interesting to note that it can already be present during adolescence. Nonetheless, there were no differences in age between patients with AN-R and AN-B/P, namely, age was not associated with the presence of shorter TLs in the AN-B/P group. Lastly, the lack of significant associations for TL with cholesterol and thyroid hormones might be related to their normal levels at both admission and discharge, likely related to the short duration of illness of our patients.

The findings of this study should be regarded as preliminary because of its limitations. First, control participants were recruited as a convenience sample and were assessed only once. They were not assessed for non-ED comorbid psychopathology, BMI-SDS, or cholesterol and thyroid hormone functioning. Second, patients with AN were significantly younger than controls, although the two AN variants were of similar ages. Third, we did not assess emotional confounders (e.g., anxiety disorders and depression) potentially associated with TL shortening in the patients with AN. Fourth, the small number of patients completing the study (*n* = 18), related to a high attrition rate, did not enable any conclusion about the effect of improvement in BMI-SDS and reduction of pathological eating behaviors on TL shortening (although we did not use scales assessing the severity of pathological eating behaviors, it is of note that patients with AN-B/P type in this department were not discharged before achieving a stabilization of their B/P behaviors). Fifth, our sample did not include male patients. Last, as the study included only inpatients, our findings cannot be generalized to ambulatory populations with less-severe AN.

A major strength of the study is that, to the best of our knowledge, it is the first to assess TL shortening in patients with AN. The group with AN was well characterized because a structured interview was used for diagnosis, and only patients whose diagnosis of AN was unanimous in the department’s clinical team meetings could enter the study.

## 5. Conclusions

The aim of this study was to assess whether the TL of female adolescents with AN was shorter than that of controls, and whether improvement in the patients’ weight and ED-related pathology was reflected in reduced TL shortening. We found that patients with AN-B/P showed significantly shorter TLs compared with patients with AN-R already at a relatively young age and after a short duration of illness. This finding was not related to differences in age or BMI-SDS. The associations found in this study between baseline B/P behaviors and TL shortening might support the importance of treating AN patients with B/P behaviors with specifically tailored therapies, e.g., cognitive behavioral or exposure interventions [65,66].

Future studies should be longitudinal and prospective, including a larger sample of ambulatory patients with AN and other types of EDs, with a wide age range, and extending over several years. Researchers should assess relevant physiological correlates of TL shortening (cognitive functioning, endocrine, metabolic, or renal disturbances) and psychological correlates (anxiety, depression) using accepted rating scales. In addition to showing differences between patients with EDs and controls regarding TL shortening, such studies might also assess the reversibility of TL shortening in EDs with the stabilization of the patients’ physical and psychological condition. We hope that our study will be a first modest step and that future studies assessing TL in EDs would build a more solid methodological basis to support our preliminary results.

## Figures and Tables

**Table 1:** Comparison between patients with anorexia nervosa (AN) at admission and controls.

	Controls (*n* = 22)	AN (*n* = 44)	*p*
	M (SD)	M (SD)	
Age in years	16.63 (1.88)	15.40 (1.54)	0.006
Telomere length (TL) in base pairs (bp)	10.74 (1.39)	9.74 (2.35)	0.62 *

Note: * results corrected by age.

**Table 2:** Comparison between patients in the two anorexia nervosa (AN) subtypes at admission.

	AN-B/P (*n* = 18)	AN-R (*n* = 26)	*p*
	M SD	M SD	
Age in years	15.78 (1.42)	15.08 (1.58)	0.14
Age at AN onset in years	13.56 (1.44)	12.87 (1.11)	0.08
Telomere Length (TL) in base pairs (bp)	8.17 (2.11)	12.26 (2.78)	0.01 *
BMI (kg/m^2^)	18.48 (1.93)	17.05 (2.24)	0.03
BMI-SDS	−0.88 (0.95)	−1.54 (1.32)	0.06
Cholesterol (mg/dL)	167.53 (21.02)	164.78 (25.27)	0.71
T4 (pmol/L)	9.87 (1.80)	9.65 (1.33)	0.64
T3 (pmol/L)	5.01 (0.85)	5.04 (0.85)	0.90
TSH (mμ/L)	2.28 (1.01)	2.57 (1.45)	0.47

Note: AN-R: anorexia nervosa restricting type; AN-B/P: anorexia nervosa binge-purge type; BMI: body mass index; BMI-SDS: body mass index standard deviation score; T4: thyroxine: T3: triiodothyronine; TSH: thyroid stimulating hormone; * results corrected by age.

**Table 3:** Comparison between admission and discharge data of 18 patients with anorexia nervosa (AN) completing the study.

	Patients with AN (*n* = 18)		*p*
	Admission	Discharge	
	M SD	M SD	
Age in years	15.24 (1.27)	15.60 (1.18)	0.36
Telomere Length (TL) in base pairs (bp)	8.62 (2.45)	8.87 (2.29)	0.36 *
BMI (kg/m^2^)	17.86 (2.53)	20.59 (1.96)	0.0005
BMI-SDS	−1.16 (1.06)	−0.14 (0.63)	0.003
Cholesterol (mg/dL)	167.94 (23.40)	175.05 (44.06)	0.53
T4 (pmol/L)	9.93 (2.03)	9.64 (1.64)	0.62
T3 (pmol/L)	5.06 (0.90)	5.60 (0.89)	0.064
TSH (mμ/L)	2.49 (1.59)	2.32 (1.01)	0.69

Note: BMI: body mass index; BMI-SDS: body mass index standard deviation score; T4: thyroxine: T3: triiodothyronine; TSH: thyroid stimulating hormone; * results corrected by age.

**Table 4:** Comparison of the patients in the two anorexia nervosa (AN) subtypes assessed at admission and discharge.

	AN-R Admission (*n* = 12)	AN-B/P Admission (*n* = 6)	*p*	AN-R Discharge (*n* = 12)	AN-B/P Discharge (*n* = 6)	*p*
	M SD	M SD		M SD	M SD	
TL	10.97 (3.91)	8.04 (4.61)	0.21	9.93 (3.41)	9.42 (3.42)	0.75
BMI	16.72 (2.30)	19.48 (1.46)	0.01	19.87 (1.88)	20.68 (1.30)	0.99
BMI-SDS	−1.59 (1.35)	−0.30 (0.49)	0.04	−0.18 (0.75)	0.06 (0.39)	0.48

Note: AN-R: anorexia nervosa restricting type; AN-B/P: anorexia nervosa binge-purge type; BMI-SDS: body mass index standard deviation score.

**Table 5:** Correlations between baseline TL and the different demographic and clinical variables introduced.

	Baseline TL	*p*
Age	r = −0.308	0.027
Age at illness onset	r = −0.411	0.113
Duration of illness at admission	r = 0.288	0.280
Baseline BMI	r = −0.124	0.384
Baseline BMI-SDS	r = −0.174	0.221
Baseline cholesterol	r = 0.120	0.423
Baseline T4	r = −0.139	0.332
Baseline T3	r = −0.149	0.301
Baseline TSH	r = 0.053	0.710

Note: BMI: body mass index; BMI-SDS: body mass index standard deviation score; TL: telomere length; T4: thyroxine; T3: triiodothyronine; TSH: thyroid stimulating hormone.

## Data Availability

The data of this study are available upon request.
